# Evaluation of a Positive Youth Development Program Based on the Repertory Grid Test

**DOI:** 10.1100/2012/372752

**Published:** 2012-04-19

**Authors:** Daniel T. L. Shek

**Affiliations:** ^1^Department of Applied Social Sciences, The Hong Kong Polytechnic University, Hong Kong; ^2^Public Policy Research Institute, The Hong Kong Polytechnic University, Hong Kong; ^3^Department of Social Work, East China Normal University, Shanghai 200062, China; ^4^Kiang Wu Nursing College of Macau, Macau; ^5^Division of Adolescent Medicine, Department of Pediatrics, Kentucky Children's Hospital, University of Kentucky College of Medicine, Lexington, KY 40506, USA

## Abstract

The repertory grid test, based on personal construct psychology, was used to evaluate the effectiveness of Project P.A.T.H.S. (Positive Adolescent Training through Holistic Social Programmes) in Hong Kong. One hundred and four program participants (*n* = 104) were randomly invited to complete a repertory grid based on personal construct theory in order to provide both quantitative and qualitative data for measuring self-identity changes after joining the program. Findings generally showed that the participants perceived that they understood themselves better and had stronger resilience after joining the program. Participants also saw themselves as closer to their ideal selves and other positive role figures (but farther away from a loser) after joining the program. This study provides additional support for the effectiveness of the Tier 1 Program of Project P.A.T.H.S. in the Chinese context. This study also shows that the repertory grid test is a useful evaluation method to measure self-identity changes in participants in positive youth development programs.

## 1. Introduction

There are growing adolescent developmental issues, such as mental health problems, abuse of psychotropic substances, adolescent suicide, school violence, and a drop in family solidarity, in Hong Kong that deserve the attention of helping professionals [1].With reference to these problems, primary prevention programs that target specific adolescent developmental problems and positive youth development programs are called for. Furthermore, attempts to identify “at risk” adolescents at an earlier stage are important. However, research findings showed that there were very few systematic and multiyear positive youth development programs in Hong Kong. Even if such programs existed, they commonly dealt with isolated problems and issues in adolescent development (i.e., deficits-oriented programs focusing on adolescent problems), and they were relatively short term in nature. In addition, systematic and long-term evaluation of the available programs did not exist [2].

To promote holistic development among adolescents in Hong Kong, the Hong Kong Jockey Club Charities Trust approved HK$400 million to launch a project entitled “P.A.T.H.S. to Adulthood: A Jockey Club Youth Enhancement Scheme.” P.A.T.H.S. denotes Positive Adolescent Training through Holistic Social Programme. The Trust invited academics of five universities in Hong Kong to form a research team, with The Hong Kong Polytechnic University as the lead institution, to develop a multiyear, universal positive youth development program to promote holistic adolescent development in Hong Kong. Besides developing the program, the research team also provides training for the teachers and social workers who implement the program and carries out longitudinal evaluation of the project [3]. Because of the overall success in the initial phase, the project was extended for another cycle of 3 years, with an additional earmarked grant of HK$350 million.

There are two tiers of programs (Tier 1 and Tier 2 Programs) in this project. The Tier 1 Program is a universal positive youth development program, where students in secondary 1 to 3 participate in the program, normally with 20 h of training in the school year at each grade. Because research findings suggested that roughly one-fifth of adolescents need help of a deeper nature, the Tier 2 Program is generally provided for at least one-fifth of the students who have greater psychosocial needs at each grade (i.e., selective program). To promote the positive development of adolescents, a total of 15 adolescent developmental constructs are covered in these two tiers of program, particularly the Tier 1 Program. These include promotion of bonding, cultivation of resilience, promotion of social competence, promotion of emotional competence, promotion of cognitive competence, promotion of behavioral competence, promotion of moral competence, cultivation of self-determination, promotion of spirituality, development of self-efficacy, development of a clear and positive identity, promotion of beliefs in the future, provision of recognition for positive behavior, provision of opportunities for prosocial involvement, and fostering prosocial norms [4].

Although there are many unique attributes of the project, the usefulness of the project remains to be demonstrated. To provide a comprehensive picture of the effectiveness of the project, several evaluation strategies are employed, including objective outcome evaluation, subjective outcome evaluation, qualitative evaluation based on focus groups, student diaries and in-depth interviews, process evaluation, and interim evaluation. To date, research findings based on different evaluation strategies demonstrated that different stakeholders held positive perceptions of the program, and the participants showed positive changes after joining the project. For example, subjective outcome evaluation consistently showed that more than four-fifths of the participants and program implementers perceived the program to be beneficial to the program participants [5–7]. Objective outcome evaluation findings also consistently showed that relative to the control participants, students in the experimental group had better positive youth development but lower levels of substance abuse and delinquency [8–10].

To further understand the effectiveness of the project, evaluation based on the approach of personal construct psychology was carried out in this study. According to Kelly [11], the universe is an ongoing process that can only be understood in terms of construction and reconstruction (e.g., from a good marriage to divorce) through the personal constructs of a person, which refer to the world views, interpretations, and deductions about life. Actually, personal constructs are transparent templates through which the external reality is understood or categories of thoughts by which an individual construes or interprets his personal world (e.g., meaningful versus not meaningful). Personal construct psychology has been used in different areas, including psychotherapy, personality assessment, organizational psychology, and education.

The repertory grid test was designed by Kelly in order to understand the personal constructs of an individual. In particular, the repertory grid test has been used to understand the self-identity system in numerous studies [12–15]. There are three unique features of the repertory grid test. First, the assessment method measures the individual's self-identity system from the perspective of the individual (i.e., using the language of the informant). This is important because the informant's own language reflects his/her subjective world view. Second, data generated from the assessment method can be analyzed via quantitative as well as qualitative methods. This attribute is desirable because there is a growing consensus that both types of data are important. Finally, the repertory grid test is a very flexible method (e.g., adjusting the number of elements and constructs) that can yield very rich information. Borell et al. [16] provided a review on the use of the repertory grid technique in the social work context. Unfortunately, this technique has not been systematically utilized in the Chinese culture in the context of evaluation.

The repertory grid method as an evaluation strategy, used to examine changes in ex-mental patients who joined a holistic psychiatric rehabilitation program conducted by Luk and Shek [17], is presented here as an illustration. The study investigated perceived personal changes in ex-mental patients after attending a psychiatric rehabilitation program in Hong Kong. The program used a self-help group approach with holistic care elements that emphasized the physical, psychological, social, and spiritual functioning of the participants. Nineteen participants were invited to complete the repertory grid test to measure self-identity changes after joining the program. Ten supplied elements and ten constructs were elicited from triads. This procedure was intended to produce two contrasting poles for the construct. The constructs were then linked to elements by a 6-point rating scale. To assess the self-identity systems of the program participants, the following elements were included: Element 1 (E1: self before joining the fellowship), Element 2 (E2: self 1 year prior), Element 3 (E3: self at present), Element 4 (E4: self 1 year later), Element 5 (E5: ideal self at present), Element 6 (E6: my father), Element 7 (E7: my mother), Element 8 (E8: one significant sibling), Element 9 (E9: an ideal ex-mental patient), and Element 10 (E10: an unsuccessful person). Regarding Element 9 (an ideal ex-mental patient), because every person has some expectations about the “ideal” conditions of a recovering (or recovered) mental patient (e.g., able to work, symptom-free, and balanced), this element can be used as the basis to compare the different selves of the participants.

With E5 (ideal self at present) as the anchoring point, the mean distances between E1–E5 and E3–E5 were 1.12 and 0.81, respectively. Analyses showed that the mean distance between E1 and E5 was significantly longer than that between E3 and E5. With the anchoring point on E9 (an ideal ex-mental patient), the mean distances between E1–E9 and E3–E9 were 1.27 and 0.85, respectively. *T*-test analysis showed that the mean distance between E1 and E9 was significantly longer than that between E3 and E9. When the anchoring point was on E10 (an unsuccessful person), the mean distances between E1–E10 and E3–E10 were 0.84 and 1.12, respectively. *T*-test analysis showed that the mean distance between E1 and E10 was significantly shorter than that between E3 and E10. The findings showed that all related statistical analyses were statistically significant. It should be noted that the findings were still significant after Bonferroni correction.

The repertory grid technique was also used to examine the effectiveness of youth drug prevention programs in Hong Kong. Shek and associates [18] utilized the repertory grid methodology to examine how the participants perceived themselves before and after joining the program. Group analyses of the grid data of the 30 informants showed that the selves of the participants improved in several areas after joining the program. First, the informants psychologically identified themselves more with the “ideal self.” Second, the informants psychologically identified themselves less with “a drug addict.” Third, the informants psychologically identified themselves more with “a successful person.” Fourth, the informants psychologically identified themselves less with “an unsuccessful person.” Further qualitative findings showed that there are significant changes in the selves of the informants after joining the program. For example, before joining the program, one informant perceived herself as playful, did not understand herself, had no future, had no goals, was a failure, was nonpersuasive, had no self-control, had no power, and did not listen to others. However, after joining the program, the informant perceived herself to have the following characteristics: maturity, understood herself, thought about the future, had goals, was successful, was persuasive, had self-control, had power, and accepted others' views. An examination of the grid data showed that before joining the program, the informant perceived herself to be very close to a drug addict and an unsuccessful person. However, after joining the program, she began to identify with the ideal self and a successful person.

Adopting the repertory grid test methodology, the present study attempted to look at how the participants perceived changes in their self-identity system after joining the Tier 1 Program of Project P.A.T.H.S. for 3 years, thus providing an additional perspective on the effectiveness of the program. The basic expectation was that the program should help the participants to identify more with the positive roles but less with the negative roles; hence, enhancing their protective factors to resist negative influences.

## 2. Methods

This study adopted the repertory grid technique originally devised by Kelly [11] in his Role Construct Repertory Test, a method of exploring a personal construct system. A self-identity system put forward by Norris and Makhlouf-Norris [19] was also adopted to measure the self-identification of the participants. By examining how a person identifies him/herself (self-identification) and the people who are significant to his/her life, the person's behaviors can be understood. One advantage of the grid method is that it can generate both quantitative and qualitative data in a single study for analysis.

A repertory grid test typically consists of elements and constructs. In the present study, there were 10 constructs elicited from triads. The triads involved selecting groups of three elements from the full list of elements, and the subject was then invited to say in what ways two of the elements were alike and in what way the third element was different from the other two. This procedure was intended to produce two contrasting poles for the construct. The constructs were then linked to elements by a 6-point rating scale. The grid was analyzed by using the INGRID package devised by Slater based on principal components analysis [20]. There are some studies examining the self-representation in the context of clinical intervention [21–24].

In the present study, a total of 108 students who had participated in the Tier 1 Program were randomly selected from the participating schools and completed the grids. However, due to missing constructs or elements in four cases, only 104 cases were used for data analysis. The following ten elements were used to elicit the constructs. Element 1: self before joining Project P.A.T.H.S. (past self), Element 2: self after joining Project P.A.T.H.S. for 3 years (present self), Element 3: self at high-school graduation (future self), Element 4: ideal self, Element 5: father, Element 6: mother, Element 7: an important friend, Element 8: a successful person, Element 9: a loser, and Element 10: a mature peer.

The constructs were elicited via the triadic method. For every triad (i.e., three elements in which a self-element was presented), the participant was asked to construct the way that two elements were most alike but were most different from the third one. Each participant was invited to elicit 10 constructs. After the constructs were elicited, the participant was then asked to rate all the elements on the construct (i.e., along the construct and contrast poles) on a 6-point scale, with 1 to 3 representing the contrast pole, and 4 to 6 representing the construct pole.As adolescents are sometimes not very verbal in their responses, two supplied constructs were added. These were “understand oneself” versus “does not understand oneself,” and “high resilience” versus “low resilience.” 

The raw data for each grid (10 elements by 12 constructs) were analyzed by the INGRID 72 program [20]. In the output of the analysis, there is a section on the distances between pairs of elements. Distances between elements represent the psychological distances with a minimum value of 0 and a mean of 1, and the value seldom exceeds 2. Therefore, if the distance between a pair of elements is close to 0, it means that they are seen as similar in terms of perceived psychological space of the person. On the other hand, if the distance between a pair of elements is close to 2, it means that they are seen to be dissimilar in the psychological space of the informant. Norris and Makhlouf-Norris [19] suggested that distances between elements generated by INGRID 72 could be used to examine how a person sees him/herself as being similar or dissimilar to certain people and others.

## 3. Hypotheses

To examine the perceived changes of the participants after joining Project P.A.T.H.S., differences in the distances between the following pairs of elements were assessed.

1. The mean distance between “self before joining Project P.A.T.H.S.” (Element 1) and “ideal self” (Element 4) versus the mean distance between “self after joining Project P.A.T.H.S.” (Element 2) and “ideal self” (Element 4): it was expected that if the participants had positive changes after joining the program, the distance between E2 and E4 would be shorter than that between E1 and E4 (i.e., the self after joining Project P.A.T.H.S. would be seen as relatively closer to the ideal self).
2. The mean distance between “self before joining Project P.A.T.H.S.” (Element 1) and “a successful person” (Element 8) versus the mean distance between “self after joining Project P.A.T.H.S.” (Element 2) and “a successful person” (Element 8): it was expected that if the participants had positive changes after joining the program, the distance between E2 and E8 would be shorter than that between E1 and E8 (i.e., the self after joining Project P.A.T.H.S. would be seen as relatively closer to a successful person).
3. The mean distance between “self before joining Project P.A.T.H.S.” (Element 1) and “a loser” (Element 9) versus the mean distance between “self after joining Project P.A.T.H.S.” (Element 2) and “a loser” (Element 9): it was expected that if the participants had positive changes after joining the program, the distance between E2 and E9 would be longer than that between E1 and E9 (i.e., the self after joining Project P.A.T.H.S. would be seen as relatively farther away from a loser).
4. The mean distance between “self before joining Project P.A.T.H.S.” (Element 1) and “a mature peer” (Element 10) versus the mean distance between “self after joining Project P.A.T.H.S.” (Element 2) and “a mature peer” (Element 10): it was expected that if the participants had positive changes after joining the program, the distance between E2 and E10 would be shorter than that between E1 and E10 (i.e., the self after joining Project P.A.T.H.S. would be seen as closer to a mature peer).

For the supplied constructs, it was expected that the self after joining Project P.A.T.H.S. (E2) would have higher mean ratings in the constructs of “understand oneself” versus “does not understand oneself,” and “high resilience” versus “low resilience.” 

## 4. Results

Repertory grid test data can be analyzed by both quantitative and qualitative methods [21–24]. For quantitative data analyses, many computer programs are available [23]. For the present study, INGRID 72 was used to analyze the data, the details of which can be seen in the work of Slater [20]. INGRID 72 generates a wide range of information, including distances between elements in the psychological space of the informant. According to Norris and Makhlouf-Norris [19], the distance between two elements can be regarded as the degree of similarity or dissimilarity between two elements, and this measure can be regarded as an indicator of a person's degree of identification with an element. Stanley [24] used distances between elements to assess psychological and social alienation in young offenders.

## 5. Group Analyses

Group analyses of the grid data of the 104 informants showed that the expectations outlined in the abovementioned hypotheses were supported.

1. The mean distance between Element 1 (self before joining Project P.A.T.H.S.) and Element 4 (ideal self) was significantly longer than the mean distance between Element 2 (self after joining Project P.A.T.H.S.) and Element 4 (ideal self): mean distance = 1.32 versus 0.88; *t*(103) = 15.801, *P* < 0.0001; effect size = 1.60. In other words, the informants psychologically identified themselves more with the “ideal self” after joining the program.
2. The mean distance between Element 1 (self before joining Project P.A.T.H.S.) and Element 8 (a successful person) was longer than the mean distance between Element 2 (self after joining Project P.A.T.H.S.) and Element 8 (a successful person): mean distance = 1.20 versus 0.90; *t*(103) = 10.325, *P* < 0.0001; effect size = 1.01. In other words, the informants psychologically identified themselves more with “a successful person” after joining the program.
3. The mean distance between Element 1 (self before joining Project P.A.T.H.S.) and Element 9 (a loser) was significantly shorter than the mean distance between Element 2 (self after joining Project P.A.T.H.S.) and Element 9 (a loser): mean  distance = 0.85 versus 1.10; *t*(103) = −9.027, *P* < 0.0001; effect size = 1.10. In other words, the informants psychologically identified themselves less with “a loser” after joining the program.
4. The mean distance between Element 1 (self before joining Project P.A.T.H.S.) and Element 10 (a mature peer) was significantly longer than the mean distance between Element 2 (self after joining Project P.A.T.H.S.) and Element 10 (a mature peer): mean distance = 1.10 versus 0.85; *t*(103) = 9.027, *P* < 0.0001; effect size = 1.01. In other words, the informants psychologically identified themselves more with “a mature peer” after joining the program.

Consistent with the hypotheses, findings showed that selves after joining Project P.A.T.H.S. were seen to be significantly different from selves before joining program. The participants saw themselves as understanding themselves after joining the program (mean = 3.01  before joining Project P.A.T.H.S., and mean = 4.44  after joining Project P.A.T.H.S.; *t*(103) = −15.333, *P* < 0.0001; effect size = 1.61). The participants also saw themselves as having a higher sense of resilience after joining the program (mean = 2.95 before joining Project P.A.T.H.S., and mean = 4.44 after joining Project P.A.T.H.S.; *t*(103) = −14.844, *P* < 0.0001; effect size = 1.57).

## 6. Individual Grid Analyses: Exemplar Cases

In addition to group analyses, data collected by repertory grid tests can also be analyzed at the individual grid level. Three exemplar cases showing drastic positive changes in the informants after joining the Project P.A.T.H.S. are presented for illustration.

**Exemplary Case 1 (Informant Number 5)**

**Figure 1:**
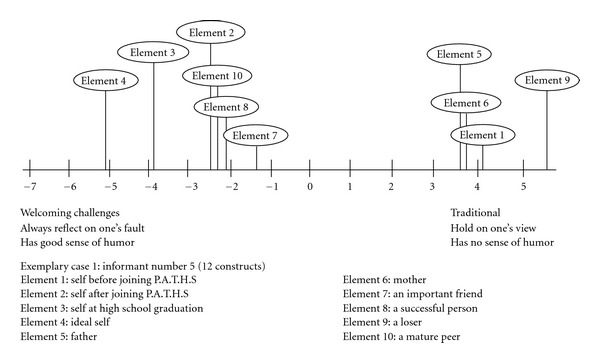
Line graph showing the mental representation of different roles for case 5.

The informant perceived herself to have the following qualities before joining Project P.A.T.H.S. (Element 1): like talking, indifferent, extrovert, care for friends, stick to established practice, hold on to one's own view, has no sense of humor, willing to share innermost feelings with others, mean, calculating, does not understand oneself, and low resilience (see Table 1). However, the informant perceived herself to have the following characteristics after joining Project P.A.T.H.S.: like talking, passionate, extrovert, care for friends, welcome challenging tasks, always reflect on one's own faults, has good sense of humor, willing to share innermost feelings with others, mean, not calculating, understand oneself, and high resilience. An examination of the grid data showed that the informant perceived herself to be very close to a loser before joining the program. However, she began to identify with the ideal self and a successful person but not with a loser after joining the program. The perceptions of the different elements in the psychological space of the informant based on the first factor of the principal components analyses are presented in Figure 1. In the line graph, the findings clearly suggest that the informant perceived that the self before and after joining Project P.A.T.H.S. was very different and the positive changes after joining program were remarkable.

**Exemplary Case 2 (Informant Number 13)**

**Figure 2:**
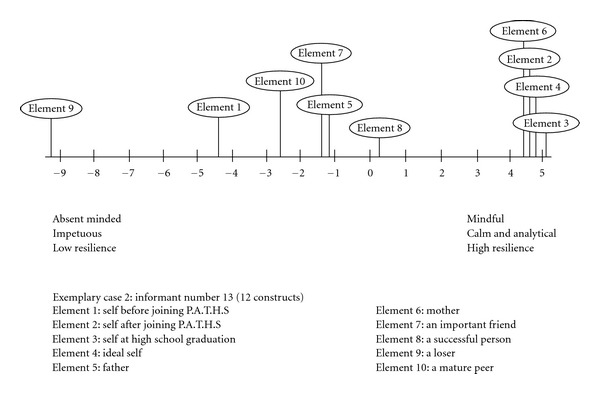
Line graph showing the mental representation of different roles for case number 13.

The informant perceived himself to have the following qualities before joining the Project P.A.T.H.S. (Element 1): absent minded, pessimistic, bad temper, complexity in thinking, impetuous, insincere, nonrestrained, flexible in thinking, direct in talking, selfish, does not understand oneself, and low resilience (see Table 2). However, the informant perceived himself to have the following characteristics after joining Project P.A.T.H.S.: mindful, optimistic, good temper, complexity in thinking, calm and analytical, sincere, restrained, flexible in thinking, direct in talking, selfless, understand oneself, and high resilience. An examination of the grid data showed that the informant perceived himself to be very close to a loser before joining the program. However, he began to identify with the ideal self and a successful person, but not with a loser after joining the program. The perceptions of the different elements in the psychological space of the informant based on the first factor of the principal components analyses are presented in Figure 2. In the line graph, the findings clearly suggest that the informant perceived that the self before and after joining Project P.A.T.H.S. was very different, and the positive changes after joining the program were remarkable.

**Exemplary Case 3 (Informant Number 67)**

**Figure 3:**
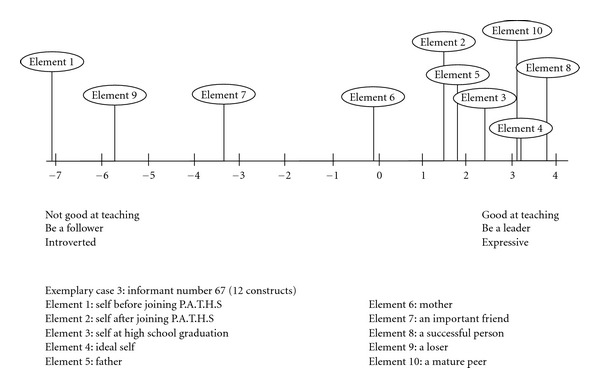
Line graph showing the mental representation of different roles for case 67.

The informant perceived himself to have the following qualities before joining Project P.A.T.H.S. (Element 1): relaxed, does not like thinking, not good at teaching, self-centered, easy going, irritable, hold on to one's own view, a follower, introverted, does not understand oneself, and high resilience (see Table 3). However, the informant perceived himself to have the following characteristics after joining Project P.A.T.H.S.: relaxed, like thinking, good at teaching, care for others, easy going, irritable, open to advice, a good leader, expressive, understand oneself, and high resilience. An examination of the grid data showed that the informant perceived himself to be very close to a loser before joining the program. However, he began to identify with the ideal self and a successful person, but not with a loser after joining the program. The perceptions of different elements in the psychological space of the informant based on the first factor of the principal components analyses are presented in Figure 3. In the line graph, the findings clearly suggest that the informant perceived that the self before and after joining Project P.A.T.H.S. was very different and the positive changes after joining the program were remarkable.

## 7. Discussion

There are several unique features of this study. First, a large sample was employed to look at the self-representation of the informants. Second, participants who joined the Tier 1 Program were randomly selected from the participating schools, thus reinforcing the generalizability of the findings. Third, both quantitative and qualitative data were collected. Fourth, this is the first known evaluation study in different Chinese communities utilizing the repertory grid technique based on a large sample size. Finally, this is also the first known scientific study utilizing the repertory grid technique in the field of positive youth development to evaluate program effectiveness using this methodology.

The evaluation findings based on the repertory grid tests clearly revealed the beneficial effects of P.A.T.H.S. Project. Using data based on 104 informants, group analyses showed that the informants identified more with the “ideal self” and “a successful person,” but less with “a loser” after joining the program. Individual analyses using three exemplar cases also illustrated the perceived positive changes in the informants after joining the program. The use of the repertory grid data provides an additional perspective to understand the program effects. Because the repertory grid technique is a “disguised” form of assessment in which the informants would find it difficult to figure out the purposes of the assessment, the chance of having demand characteristics in the assessment process was not high. As such, the positive findings point to the beneficial effects of the program. In particular, the increased distance between the “present self” and the “loser” suggests that the informants were less motivated to identify with the loser. Because the repertory grid techniques have been rarely used in the evaluation of positive youth development programs, the present attempt is a pioneering effort in the literature. If resources permit, it is recommended that this method should be used to assess the perceived self-identity systems of all program participants.

One unique characteristic of Project P.A.T.H.S. is the systematic evaluation of the program. A wide range of evaluation strategies have been used to evaluate the Tier 1 Program as follows;

1. *Objective outcome evaluation*: a randomized group trial with 24 experimental schools and 24 control schools initially has been carried out.
2. *Subjective outcome evaluation (Tier 1 Program)*: both students and program implementers are invited to complete subjective outcome evaluation forms (form A and form B, resp.) after completion of the program.
3. *Process evaluation*: systematic observations are carried out in randomly selected schools to understand the program implementation details.
4. *Interim evaluation*: to understand the process of implementation, interim evaluation is conducted by randomly selecting roughly half of the participating schools in the experimental and full implementation phases.
5. *Qualitative evaluation (focus groups based on students)*: focus groups involving students based on schools randomly selected from the participating schools are carried out.
6. *Qualitative evaluation (focus groups based on program implementers)*: focus groups involving instructors based on schools randomly selected from the participating schools are carried out.
7. *Qualitative evaluation (in-depth interviews with program implementers)*: Prolonged in-depth interviews with teachers are carried out.
8. *Qualitative evaluation (case study based on focus groups)*: case studies documenting the implementation experience of schools that have incorporated the Tier 1 Program into school formal curriculum are carried out.
9. *Qualitative evaluation (student logs)*: students are invited to reflect on their experiences after attending P.A.T.H.S. lessons and to apply things learned to their real lives.
10. *Qualitative evaluation (student products)*: students' weekly diaries are collected after completion of the program. Students' drawings are also collected to reflect the experiences of the program participants.
11. *Management information collected from the cowalker scheme*: Because the cowalkers conducted classroom observations and completed observation forms, such information can give an overall picture about the implementation details in different schools.
12. *Evaluation based on the repertory grid tests*: students are randomly selected to complete repertory grid tests that assess their self-identity systems before and after joining the program and perceived changes across years.

Borrowing concepts from navigation and military disciplines, Denzin [25] used the term “triangulation” to argue for the utilization of different types of data based on different methodologies to examine the same phenomenon. The basic belief underlying the concept of triangulation is that there are biases in any one type of investigation, and such biases and errors would be revealed and cancelled out when different methods, data sources, and/or investigators are involved. In other words, triangulation refers to the process of seeking convergence of results based on different methods, researchers, and settings on the same phenomenon under observation. Evaluators generally suggest that triangulation is an important principle that should be utilized to check the quality of evaluation data. Generally speaking, triangulation of the available evaluation findings shows that different stakeholders had positive views about the Tier 1 Program, and they perceived the program to be beneficial to the development of the program participants. Most importantly, the findings suggest that the project is effective in promoting positive youth development among Chinese adolescents in Hong Kong [26–28].

The present study underscores the utility of using the repertory grid test to assess changes in program participants. According to some researchers, the repertory grid technique can provide data from the informants' own views and thus represents a better method than questionnaire-type assessments, which may elicit only socially desirable responses. With some exceptions, the use of the repertory grid technique is not common in social work or related professions in Hong Kong. Because of the wide applicability of the repertory grid technique in clinical settings [11], helping professionals should consider using it to assess changes in participants joining prevention programs.

Of course, it is noteworthy that there are several limitations of the study. First, although a representative sample used in this study may permit generalizability of the findings, replication of the findings is necessary. In particular, it would be exciting to look at the prolonged changes in a person over time. Second, although statistically significant findings were obtained, it should be noted that because of the retrospective nature of the design (i.e., present construction of past roles), the clinical significance of the findings cannot be easily assessed. As such, data collected in real-life contexts would be helpful. Third, because there was no comparison group in the study, there is the possibility that the perceived changes in the participants can be attributed to factors other than holistic intervention (e.g., natural maturation changes). Finally, because no manipulation of the independent variable was carried out, the design does not permit us to draw the conclusion that the positive youth development program caused positive changes in the informants. Of course, the counterargument to this view is that looking at the self-representation from the perspective of the informants may give a clearer and more comprehensive picture about the beneficial effects of the Tier 1 Program of Project P.A.T.H.S. in Hong Kong.

## Figures and Tables

**Table 1:** Raw grid form for case no. 5.

	(1)	(2)	(3)	(4)	(5)	(6)	(7)	(8)	(9)	(10)	Construct pole	Contrast pole
	Self before joining Project P.A.T.H.S. (past self)	Self after joining Project P.A.T.H.S. for 3 years (present self)	Self at high school graduation (future self)	Ideal self	Father	Mother	An important friend	A successful person	A loser	A mature peer	6 5 4	3 2 1
												
1	**6**	**5**	**5**	**5**	**2**	**5**	**4**	**5**	**6**	**5**	Like talking	Does not like talking
2	**3**	**5**	**5**	**5**	**2**	**4**	**5**	**4**	**4**	**5**	Passionate	Indifferent
3	**4**	**4**	**2**	**2**	**5**	**5**	**2**	**2**	**5**	**3**	Extrovert (catch others' eyes)	Introvert
4	**4**	**6**	**5**	**5**	**2**	**3**	**5**	**4**	**5**	**5**	Care for friends	Self-centered
5	**2**	**5**	**5**	**6**	**3**	**1**	**3**	**5**	**2**	**6**	Welcome challenging tasks	Stick to established practice
6	**2**	**6**	**6**	**6**	**3**	**3**	**5**	**5**	**2**	**5**	Always reflect on one's own faults	Hold on to one's own view
7	**3**	**5**	**5**	**6**	**3**	**1**	**4**	**5**	**1**	**4**	Has good sense of humor	Has no sense of humor
8	**4**	**6**	**6**	**6**	**3**	**6**	**5**	**4**	**2**	**5**	Willing to share innermost feelings with others	Introverted
9	**2**	**3**	**5**	**5**	**3**	**3**	**5**	**4**	**4**	**5**	Generous	Mean
10	**2**	**5**	**5**	**5**	**4**	**5**	**5**	**5**	**2**	**5**	Not calculating	Calculating
11	**3**	**4**	**5**	**6**	**2**	**2**	**4**	**4**	**1**	**4**	Understand oneself	Does not understand oneself
12	**3**	**5**	**6**	**6**	**5**	**5**	**5**	**6**	**3**	**5**	High resilience	Low resilience

**Table 2:** Raw grid form for case no. 13.

	(1)	(2)	(3)	(4)	(5)	(6)	(7)	(8)	(9)	(10)	Construct pole	Contrast pole
	Self before joining Project P.A.T.H.S. (past self)	Self after joining Project P.A.T.H.S. for 3 years (present self)	Self at high school graduation (future self)	Ideal self	Father	Mother	An important friend	A successful person	A loser	A mature peer	6 5 4	3 2 1
												
1	**2**	**6**	**6**	**6**	**2**	**6**	**4**	**3**	**1**	**4**	Mindful	Absent minded
2	**3**	**5**	**5**	**4**	**6**	**6**	**4**	**5**	**1**	**2**	Optimistic	Pessimistic
3	**2**	**6**	**6**	**6**	**5**	**5**	**5**	**5**	**1**	**3**	Good temper	Bad temper
4	**4**	**6**	**6**	**5**	**2**	**6**	**6**	**3**	**4**	**6**	Complexity in thinking	Simple minded
5	**2**	**5**	**6**	**6**	**4**	**5**	**3**	**4**	**1**	**1**	Calm and analytical	Impetuous
6	**3**	**6**	**6**	**6**	**4**	**6**	**5**	**5**	**1**	**4**	Sincere	Insincere
7	**2**	**5**	**5**	**5**	**1**	**6**	**2**	**3**	**1**	**3**	Restrained	Nonrestrained
8	**5**	**6**	**6**	**6**	**4**	**5**	**5**	**3**	**2**	**5**	Flexible in thinking	Rigid in thinking
9	**3**	**2**	**1**	**3**	**3**	**5**	**5**	**5**	**3**	**5**	Tactful in talking	Direct in talking
10	**3**	**5**	**5**	**5**	**4**	**6**	**5**	**5**	**1**	**3**	Selfless	Selfish
11	**3**	**5**	**5**	**6**	**4**	**5**	**3**	**5**	**1**	**5**	Understand oneself	Does not understand oneself
12	**3**	**6**	**6**	**6**	**5**	**5**	**1**	**5**	**1**	**3**	High resilience	Low resilience

**Table 3:** Raw grid form for case no. 67.

	(1)	(2)	(3)	(4)	(5)	(6)	(7)	(8)	(9)	(10)	Construct pole			Contrast pole		
	Self before joining Project P.A.T.H.S. (past self)	Self after joining Project P.A.T.H.S. for 3 years (present self)	Self at high school graduation (future self)	Ideal self	Father	Mother	An important friend	A successful person	A loser	A mature peer	6 5 4			3 2 1		
										
1	**1**	**2**	**2**	**2**	**6**	**4**	**3**	**4**	**2**	**5**	Tense (handling things)			Relaxed		
2	**2**	**4**	**5**	**5**	**4**	**3**	**2**	**4**	**2**	**4**	Like thinking			Does not like thinking		
3	**2**	**5**	**5**	**5**	**3**	**4**	**2**	**6**	**1**	**4**	Good at teaching			Not good at teaching		
4	**2**	**5**	**5**	**5**	**3**	**4**	**2**	**6**	**1**	**4**	Good at teaching			Not good at teaching		
5	**2**	**5**	**5**	**6**	**5**	**3**	**4**	**5**	**2**	**6**	Care for others			Self-centered		
6	**1**	**2**	**2**	**3**	**6**	**4**	**3**	**4**	**2**	**5**	Stubborn			Easy going		
7	**1**	**3**	**4**	**4**	**2**	**5**	**4**	**5**	**5**	**3**	Patient			Irritable		
8	**1**	**4**	**4**	**4**	**2**	**4**	**4**	**5**	**2**	**4**	Open to advice			Hold on to one's own view		
9	**1**	**5**	**6**	**6**	**5**	**3**	**2**	**5**	**1**	**6**	A good leader			A follower		
10	**5**	**2**	**2**	**2**	**1**	**3**	**4**	**2**	**4**	**2**	Introverted			Expressive		
11	**1**	**4**	**4**	**5**	**5**	**5**	**3**	**5**	**4**	**5**	Understand oneself			Does not understand oneself		
12	**4**	**4**	**5**	**5**	**3**	**4**	**3**	**4**	**5**	**4**	High resilience			Low resilience		
